# Clinical muscle mass-related biomarkers that predict mortality in older patients with community-acquired pneumonia

**DOI:** 10.1186/s12877-022-03626-y

**Published:** 2022-11-19

**Authors:** Sha Huang, Yan Guo, Lanlan Chen, Yan Wang, Xiaoyan Chen

**Affiliations:** Zigong Affiliated Hospital of Southwest Medical University, Zigong Psychiatric Research Center, Zigong, China

**Keywords:** AST/ALT, creatinine/cystatin C, sarcopenia, pneumonia, mortality

## Abstract

**Objective:**

Community-acquired pneumonia (CAP) is associated with elevated morbidity and mortality, and it usually occurs in older adults. Our goal here was to assess the efficacies of muscle mass-related biomarkers, such as, aspartate transaminase/alanine transaminase (AST/ALT) and creatinine/cystatin C*100 (Cr/CysC*100), in predicting 1-, 2-, and 3-year mortalities of older CAP patients.

**Methods:**

Design: Retrospective cohort study. Setting and Participants: A teaching hospital in western China. Hospitalized CAP patients, aged≥60 years. We separated patients into a high or low muscle mass group, according to the median AST/ALT and Cr/CysC*100, respectively. We acquired data from medical records and local government mortality databases, as well as telephonic interviews. We analyzed the association between low muscle mass (AST/ALT and Cr/CysC*100) and all-cause mortality at 1, 2, and 3 years in older patients with CAP.

**Results:**

We enrolled 606 patients (58.58% male; median age: 81 years) for analysis. The 1-, 2-, and 3-year mortality in older patients with CAP in the low muscle mass group (AST/ALT) was higher than in the high muscle mass group (AST/ALT) (1-year: 51.16% vs. 36.96%, *p* < 0.001; 2-year: 54.46% vs. 41.25%, *p* = 0.001; 3-year: 54.79% vs. 42.9%, *p* = 0.003). Upon adjustment of potential confounding factors, we revealed, using cox regression analysis, that the low muscle mass group (AST/ALT) experienced enhanced mortality risk at the 1-, 2-, and 3-year follow-ups, compared to the high muscle mass group (AST/ALT) (1-year: hazard ratios (HR) = 1.46, 95% confidence interval (CI): 1.13–1.88; 2-year: HR = 1.39, 95% CI: 1.09–1.77; 3-year: HR = 1.35, 95% CI: 1.06–1.72). The 1-, 2-, and 3-year mortality of older CAP patients in the low muscle mass group (Cr/CysC*100) was also higher than the high muscle mass group (Cr/CysC*100) (1-year: 56.29% vs. 31.91%, *p* < 0.001; 2-year: 60.26% vs. 35.53%, *p* < 0.001; 3-year: 61.26% vs. 36.51%, *p* < 0.001). Compared to the high muscle mass group (Cr/CysC*100), the low muscle mass group (Cr/CysC*100) experienced enhanced mortality risk at the 1-, 2-, and 3-year follow ups (1-year: HR = 1.9, 95% CI: 1.46–2.48; 2-year: HR = 1.85, 95% CI: 1.44–2.39; 3-year: HR = 1.85, 95% CI: 1.44–2.37).

**Conclusions:**

Low muscle mass (AST/ALT and Cr/CysC*100) were associated with enhanced 1-, 2-, and 3-year mortality risk in older patients with CAP.

**Supplementary Information:**

The online version contains supplementary material available at 10.1186/s12877-022-03626-y.

## Background

Community acquired pneumonia (CAP) has a remarkably high prevalence rate (5–10 cases/1000 individuals) and is age-dependent (more incidences in older versus young individuals) [1]. Approximately 22–42% of adult CAP patients are hospitalized, among whom about 1.2–10% require stay at the intensive care unit (ICU) [1]. CAP is the leading infectious disease in the world, and it carries an elevated mortality rate [2]. In the UK, the reported mortality rate of hospitalized adult CAP patients is between 5.7–14 %[1]. Moreover, the mortality rate among severe CAP patients requiring ICU admission in the UK is > 30% [1]. CAP is also has markedly elevated long-term mortality rates, accounting for as much as 20.8% mortality by 1 year, 34.1% by 901 days, and 35.8% by 5 years [3].

Altuna-Venegas et al. reported substantially elevated CAP prevalence in sarcopenic patients [4]. In fact, sarcopenia involving the lower paraspinal muscle area (PMA) is correlated with a markedly enhanced mortality rate in severe CAP patients in the ICU [5]. Hospitalized low-PMA patients are reported to experience a significantly reduced median overall survival (OS) duration (20.00 vs. 51.00 days, *p* < 0.001) [5]. Reduced muscle mass is a possible indicator of long-term mortality in patients with pneumonia [6]. In fact, pneumonia is correlated with one of the criteria for sarcopenia, namely, reduced grip strength, which indicates a generalized loss of muscle strength, which can also affect respiratory and oropharyngeal muscles [7]. Moreover, this syndrome is a risk factor for dysphagia [8], which is correlated with hospital readmission for aspiration- and non-aspiration-related pneumonia [9]. Therefore, early identification and intervention of sarcopenia in older CAP patients is very crucial.

Sarcopenia is an aging disease that is associated with reduced muscle mass and strength or loss of function [10]. Sarcopenia diagnosis often involves objective recordings of muscle mass, muscle strength, and physical performance. At present, dual-energy X-ray absorptiometry (DXA), and bioelectrical impedance analysis (BIA) are the most prevalent assessment strategies for appendicular skeletal muscle mass measurement in Asia [11]. Handgrip strength is also commonly used to assess skeletal muscle strength [11]. However, the above measurement methods require professional and expensive instruments. Moreover, physical performance indicators like short physical performance battery (SPPB), 6-min walk test, and stair climb power test (SCPT) are not readily available for certain older individuals. Recently, the serum creatinine/cystatin C*100 (Cr/CysC*100) was proposed as an alternative to the current sarcopenia assessment [12, 13]. Yin et al. reported that the aspartate transaminase/alanine transaminase (AST/ALT) ratio (AUC = 0.682) offered the optimal prediction of sarcopenia, among all bioindicators [14]. Clinical laboratory biomarkers, namely, AST/ALT and Cr/CysC*100, are widely used in clinical practice, and are even used for the health check of older individuals with other diseases. This is highly beneficial due to its remarkable prediction accuracy, without incurring radiation risk or increasing medical expenditure. However, there is no current information on whether AST/ALT and Cr/CysC*100 can be used as biomarkers for muscle assessment in CAP patients to predict their 1-, 2-, and 3-year mortality. Here, we explored the effectiveness of AST/ALT and Cr/CysC*100 in predicting the 1-, 2-, and 3-year mortality of older patients with CAP. Our work will provide some insights into the prognosis estimation of CAP patients for medical reference.

## Methods

### Research design and patients

The present retrospective observational study was conducted at a teaching hospital in western China between January 2016 and March 2021. The study included hospitalized CAP patients, aged ≥60 years. Specifically, we included inpatients aged ≥60 years, whose discharge diagnoses matched the relevant codes for CAP in ICD-10. The ICD-10 codes included J18.900, J98.414, J18.903, J15.903, J15.902, J15.900, J69.001, J18.200, j18.100, and j18.800 × 003. Additionally, our exclusion criteria included estimated glomerular filtration rates (eGFR) < 15 ml/min/1.73 m^2^, ALT ≥40 U/L, inaccurate patient contact details, or patients who refused to participate in clinical follow-up. An anonymous medical database was independently used by two researchers (SH and YG), who retrieved health-related variables for this study.

### Ethics

The Center for Health Informatics anonymized all relevant data, and reviewed the study protocol for this retrospective medical records-based investigation. Data confidentiality was maintained at all times, and our research followed the guidelines of the Declaration of Helsinki. Being retrospective in nature, this research did not require informed patient consent. Finally, we received approval for this study from our Research Ethics Committee (No. 2021-06-01).

### Data collection

We extracted patient demographics, such as, age, sex, alcohol and smoking habit, height, weight, systolic blood pressure (SBP), diastolic blood pressure (DBP), respiratory rate, chronic diseases (i.e., hypertension, diabetes, coronary heart disease (CHD), arrhythmia, chronic obstructive pulmonary disease (COPD), bronchial asthma, bronchiectasis, tumor, history of stroke), glasgow coma scale (GCS) score, and blood test upon admission. Information on survival or death (all-cause mortality) was collected from local governments and telephone interviews between July 1, 2021 and July 7, 2021. OS was defined as the duration between discharge and death or the last day a patient was recorded as alive.

### Clinical laboratory biomarkers

Fasting venous blood (after an overnight fast) was drawn by an experienced geriatric nurse. Two blood markers, AST/ALT and Cr/CysC*100, were used to define muscle mass. The grouping method was similar to previous articles published by our research group (that is, grouping with the median as the cutoff value) [15]. Specifically, the median of AST/ALT was 1.48, AST/ALT≤1.48 represented the high muscle mass group, AST/ALT> 1.48 represented the low muscle mass group. Similarly, the median of Cr/CysC*100 was 64.64, Cr/CysC*100 > 64.64 represented the high muscle mass group, and Cr/CysC*100 ≤ 64.64 represented the low muscle mass group.

### Statistical analysis

SPSS 25.0 was employed for all data analyses. *P* < 0.05 was the significance threshold, and all tests were two-sided. Continuous variables are expressed as mean plus or minus standard deviation or median, depending on whether they conformed to a normal distribution or not; categorical variables are presented as numbers (percentages), and the chi-square test was used to analyze whether there was any difference between the two groups. Cox proportional hazards models were used to determine the hazard ratios (HR) associated with low muscle mass (AST/ALT and Cr/CysC*100) and all-cause mortality. The multivariate Cox regression model was normalized against patient age, sex, smoking and drinking habit, BMI, number of chronic diseases, GCS score, blood urea nitrogen, SBP, DBP, and breathing rate.

## Results

Among the 696 patients enrolled in this study, 90 were eliminated as they were lost to follow-up. The remaining 606 older CAP patient information (58.58% male; median age: 81 years) was used in our final analysis (Table 1). We compared the main demographics of included patients with those lost to follow-up. Marked statistical differences existed between the included and lost to follow-up patients in terms of age, sex, smoking history, and drinking history (see Additional file 1 Table S1 for details).

**Table 1:** Baseline characteristics of participants

Variable	Total *N* = 606
Age, year, Median	81
< 80, n (%)	282(46.53)
≥ 80, n (%)	324(53.47)
Sex, n (%)	
male	355(58.58)
female	251(41.42)
Smoking history, n (%)	
no	392(65.01)
yes	211(34.99)
Drinking history, n (%)	
no	473(78.57)
yes	129(21.43)
BMI, kg/m^2^, n (%)	
< 24	517(85.45)
≥ 24	88(14.55)
Number of chronic diseases, Mean ± SD	1.97 ± 1.31
GCS score, median	15
Blood urea nitrogen, median	6
SBP, mmHg, n (%)	
≥ 90	595(98.18)
< 90	11(1.82)
DBP, mmHg, n (%)	
> 60	514(84.82)
≤ 60	92(15.18)
Breathing rate, n (%)	
< 30	590(97.36)
≥ 30	116(2.64)
AST/ALT, median	1.48
Cr/ CysC*100, Mean ± SD	64.13 ± 18.89

Note: BMI: body mass index; GCS: glasgow coma scale; SBP: systolic blood pressure; DBP: diastolic blood pressure; AST: aspartate transaminase; ALT: alanine transaminase; Cr: creatinine; CysC: Cystatin C. Comorbidities include: diabetes, hypertension, coronary heart disease (CHD), arrhythmia, chronic obstructive pulmonary disease (COPD), bronchial asthma, bronchiectasis, tumor, history of stroke

**Fig 1:**
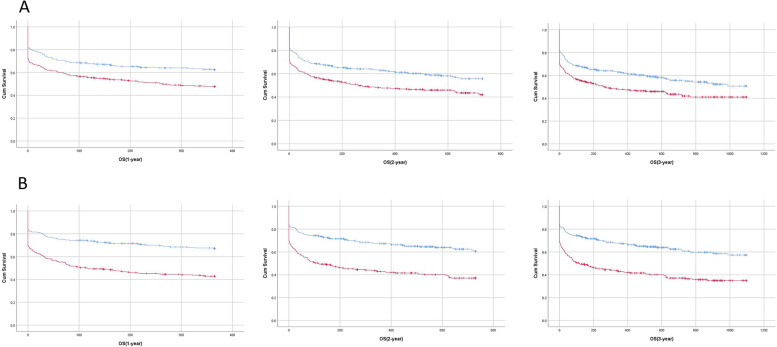
Comparision of survival time between high muscle mass group and low muscle mass group. Note: A: AST/ALT; B: Cr/CysC*100; Blue: high muscle mass; Red: low muscle mass; P values of the above 6 groups were all < 0.05

Compared to the high muscle mass group (AST/ALT), the low muscle mass group (AST/ALT) exhibited higher mortality at the 1-, 2-, and 3–year mark, and the difference was statistically significant (1 year: 51.16% vs. 36.96%, *p* < 0.001; 2 year: 54.46% vs. 41.25%, *p* = 0.001; 3 year: 54.79% vs. 42.9%, *p* = 0.003; Table 2). Compared to the high muscle mass group (Cr/CysC*100), the low muscle mass group (Cr/CysC*100) displayed higher mortality at the 1-, 2-, and 3-year mark, and the difference was statistically significant (1 year: 56.29% vs. 31.91%, *p* < 0.001; 2 year: 60.26% vs. 35.53%, *p* < 0.001; 3 year: 61.26% vs. 36.51%, *p* < 0.001; Table 2). Relative to the high muscle mass group (AST/ALT and Cr/CysC*100), the low muscle mass group (AST/ALT and Cr/CysC*100) exhibited a shorter 1-year, 2-year, and 3-year survival time (all *p* < 0.05, Figure 1).

**Table 2:** Death data of participants

Survival status	Total *N* = 606	High muscle mass (AST/ALT)	Low muscle mass (AST/ALT)	*P*-value	High muscle mass (Cr/CysC*100)	Low muscle mass (Cr/CysC*100)	*P*-value
1 year, n (%)				< 0.001			< 0.001
survival	339(55.94)	191(63.04)	148(48.84)		207(68.09)	132(43.71)	
death	267(44.06)	112(36.96)	155(51.16)		97(31.91)	170(56.29)	
2 years, n (%)				0.001			< 0.001
survival	316(52.15)	178(58.75)	138(45.54)		196(64.47)	120(39.74)	
death	290(47.85)	125(41.25)	165(54.46)		108(35.53)	182(60.26)	
3 years, n (%)				0.003			< 0.001
survival	310(51.16)	173(57.1)	137(45.21)		193(63.49)	117(38.74)	
death	296(48.84)	130(42.9)	166(54.79)		111(36.51)	185(61.26)	

Note: high muscle mass: AST/ALT≤1.48 or Cr/CysC*100 > 64.64, low muscle mass: AST/ALT> 1.48 or Cr/CysC*100 ≤ 64.64

Compared to the high muscle mass group (AST/ALT), the low muscle mass group (AST/ALT) experienced enhanced mortality risk at the 1-, 2-, and 3-year time points (1 year: HR = 1.52, 95% confidence interval (CI): 1.19–1.93; 2 year: HR = 1.46, 95% CI: 1.16–1.85; 3 year: HR = 1.42, 95% CI: 1.13–1.79; Table 3). Moreover, upon adjustment of possible confounding factors, the 1-, 2-, and 3-year patient mortality risk was still increased in the low muscle mass group (AST/ALT) (1 year: HR = 1.46, 95% CI: 1.13–1.88; 2 year: HR = 1.39, 95% CI: 1.09–1.77; 3 year: HR = 1.35, 95% CI: 1.06–1.72; Table 3). Similarly, compared to the high muscle mass group (Cr/CysC*100), the low muscle mass group (Cr/CysC*100) experienced a increases in the 1-, 2-, and 3-year mortality risks (1 year: HR = 2.05, 95% CI: 1.6–2.64; 2 year: HR = 2, 95% CI: 1.56–2.54; 3 year: HR = 1.98, 95% CI: 1.57–2.51; Table 3). Lastly, after adjusting for potential confounders, the low muscle mass group (Cr/CysC*100) still experienced augmented mortality risk at the 1-, 2-, and 3-year time points (1 year: HR = 1.9, 95% CI: 1.46–2.48; 2 year: HR = 1.85, 95% CI: 1.44–2.39; 3 year: HR = 1.85, 95% CI: 1.44–2.37; Table 3).

**Table 3:** Correlations between AST/ALT with Cr/CysC*100 and mortality

Variable	1 year mortality		2 years mortality		3 years mortality	
	P-value	HR (95% *CI*)	P-value	HR (95% *CI*)	P-value	HR (95% *CI*)
AST/ALT						
Model 1						
High muscle mass	–	1	–	1	–	1
Low muscle mass	0.001	1.52(1.19–1.93)	0.001	1.46(1.16–1.85)	0.003	1.42(1.13–1.79)
AST/ALT						
Model 2						
High muscle mass	–	1	–	1	–	1
Low muscle mass	0.03	1.46(1.13–1.88)	0.008	1.39(1.09–1.77)	0.04	1.35(1.06–1.72)
Cr/ CysC*100						
Model 1						
High muscle mass	–	1	–	1	–	1
Low muscle mass	< 0.001	2.05(1.6–2.64)	< 0.001	2(1.56–2.54)	< 0.001	1.98(1.57–2.51)
Cr/ CysC*100						
Model 2						
High muscle mass	–	1	–	1	–	1
Low muscle mass	< 0.001	1.9(1.46–2.48)	< 0.001	1.85(1.44–2.39)	< 0.001	1.85(1.44–2.37)

Note: Model 1: a non-adjusted model. Model 2: adjusting for age, sex, smoking history, drinking history, BMI, number of chronic diseases, GCS score, blood urea nitrogen, SBP, DBP, breathing rate. High muscle mass: AST/ALT≤1.48 or Cr/CysC*100 > 64.64, low muscle mass: AST/ALT> 1.48 or Cr/CysC*100 ≤ 64.64

## Discussion

In this study, we consecutively included 606 older CAP participants, aged ≥60 years, and used their clinical laboratory biomarker information, namely, AST/ALT and Cr/CysC*100, instead of muscle mass, to predict the 1-, 2-, and 3-year mortalities. To our knowledge, this is the first study that employed AST/ALT and Cr/CysC*100 to predict all-cause mortality in older CAP patients. Moreover, our study also demonstrated that the AST/ALT ratio and Cr/CysC*100 can predict the 1-year, 2-year and 3-year mortality of CAP patients. To a certain extent, it is reasonable to consider AST/ALT and Cr/CysC*100, in place of muscle mass, as prognostic indicators of older CAP mortality. Early sarcopenia detection during geriatric CAP risk assessment may facilitate early and appropriate treatment and intervention planning for geriatric CAP patients.

Maeda and colleagues previously reported that high AST/ALT is independently associated with an elevated 1-year mortality risk in hospitalized patients with heart failure. Consistent with our study. Maeda and his colleagues also revealed that AST/ALT is more strongly correlated with the 1-year mortality, independent of other covariates, other than ALT alone. This is likely because the AST and ALT proportion may offset the influence from liver dysfunction, thereby making AST/ALT more specific to muscle capacity [16]. ALT is a key enzyme in gluconeogenesis, whereby muscle protein is broken down to amino acids, which, in turn, converts alanine to alpha-ketoglutarate for glucose and/or energy production. Chung et al. proposed that low ALT represents low muscle strength [17]. In contrast, high AST with normal or low ALT primarily reflects mild to moderate skeletal muscle pathology [18]. Therefore, the AST to ALT ratio can serve as a good predictor of sarcopenia in older individuals.

Prior investigations revealed that serum Cr is strongly correlated with muscle mass [19]. Serum CysC is a low molecular weight protein. It has a stable yield, and is readily filtered by the glomerulus [11]. Sun et al. reported that the serum Cr/CysC ratio is a suitable sarcopenia indicator, compared to serum Cr, CysC [20]. Hence, the Cr/CysC*100 value, obtained from the calibration of Cr with CysC, that is independent of muscle mass, can estimate sarcopenia.

The clinical laboratory biomarkers employed in this study are part of the routine blood testing, and are, therefore, inexpensive and globally available to any hospitalized patients, with comparable reproducibility. Confirmation of low muscle mass (AST/ALT, Cr/CysC*100) during hospitalization in older pneumonia patients can enable attending physicians to make better clinical/therapeutic decisions (duration and type of antibiotics, promotion of physical therapy, and rehabilitation planning prior to patient discharge) to support optimal patient care.

## Limitation

Our work encountered certain limitations. Our investigation involved a single institution, with a relatively small population size, and it was a retrospective study, with potential selection bias. It is important to note that we only included hospitalized older adults with CAP. Compared to outpatients, older hospitalized CAP patients may be more severely ill, may be more likely to be immobilized and malnourished, may have an enhanced sarcopenia risk, and may have an enhanced mortality risk [1, 21, 22]. We excluded people who were lost to follow-up, which may have generated certain bias. Moreover, the pneumonia diagnosis was based on the patient’s discharge diagnosis, and we did not examine each patient’s pneumonia diagnosis in the electronic medical record system one by one. In addition, this investigation only included an exclusively Chinese population. Hence, its conclusions may not be applicable toward people of other races or countries. Finally, we did not employ DXA or BIA to determine the actual muscle mass. Therefore, we recommend additional prospective investigations, involving a large patient population, to confirm the results of this study.

## Conclusions

Low muscle mass (AST/ALT and Cr/CysC*100) is associated with enhanced 1-, 2-, and 3-year mortality risks in older patients with CAP. Based on our analyses, both AST/ALT and Cr/CysC*100 can serve as clinical laboratory biomarkers, instead of muscle mass determination, to predict the mortality in CAP patients.

### Availability of date and materials

The datasets generated and analyzed during the current study are not publicly available due to this is a database which has a lot of important information and we are applying some important projects based on this. But this data sets will be available 2 years later and is also available now from the corresponding author on a reasonable request.

### Funding statement

This work was funded by the 2021 Key Science and Technology Plan of Zigong City (Project No. 2021YXY12) and the 2021 Key Science and Technology Plan of Zigong City (Project No. 2021YXY07).

## Supplementary Information

**Additional file 1.** Additional file

## Abbreviations

CAP: community-acquired pneumonia
ICU: intensive care unit
PMA: paraspinal muscle area
OS: overall survival
DXA: dual-energy X-ray absorptiometry
BIA: bioelectrical impedance analysis
SPPB: short physical performance battery
SCPT: stair climb power test
AST: aspartate transaminase
ALT: alanine transaminase
Cr: creatinine
CysC: cystatin-C
eGFR: estimated glomerular filtration rates
SBP: systolic blood pressure
DBP: diastolic blood pressure
CHD: coronary heart disease
COPD: chronic obstructive pulmonary disease
GCS: glasgow coma scale
CI: confidence interval
HR: hazard ratio.
