# Supplementary material for: Clinical muscle mass-related biomarkers that predict mortality in older patients with community-acquired pneumonia
Source: BMC Geriatr. 2022 Nov 19;22:880. doi: 10.1186/s12877-022-03626-y (PMC9675073; doi:10.1186/s12877-022-03626-y)
Supplement: Supplementary file 1 — Additional file 1. Additional file [file 12877_2022_3626_MOESM1_ESM.docx]

**Table S1 Key demographic comparisons of included patients versus those lost to follow-up**

| Variable | Include  N=606 | Exclude  N=90 | P |
| --- | --- | --- | --- |
| **Age, year, Median** | 81 | 75 | 0.011 |
| **Sex, n (%)** |  |  | 0.039 |
| male | 355(58.58) | 63(70) |  |
| female | 251(41.42) | 27(30) |  |
| **Smoking history, n (%)** |  |  | 0.006 |
| no | 392(65.01) | 45(50) |  |
| yes | 211(34.99) | 45(50) |  |
| **Drinking history, n (%)** |  |  | 0.023 |
| no | 473(78.57) | 61(67.78) |  |
| yes | 129(21.43) | 29(32.22) |  |
| **BMI, kg/m^2^, n (%)** |  |  | 0.301 |
| <24 | 517(85.45) | 73(81.11) |  |
| ≥24 | 88(14.55) | 17(18.89) |  |
| **Number of chronic diseases, Mean ± SD** | 1.97±1.31 | 1.86±1.18 | 0.433 |
| **GCS score, median** | 15 | 15 | 0.474 |
| **Blood urea nitrogen, median** | 6 | 5.63 | 0.115 |
| **SBP, mmHg, n (%)** |  |  | 0.631 |
| ≥90 | 595(98.18) | 89（98.9） |  |
| <90 | 11(1.82) | 1（1.1） |  |
| **DBP, mmHg, n (%)** |  |  | 0.132 |
| >60 | 514(84.82) | 84（93.33） |  |
| ≤60 | 92(15.18) | 6（6.67） |  |
| **Breathing rate, n (%)** |  |  | 0.816 |
| <30 | 590(97.36) | 88（97.78） |  |
| ≥30 | 116(2.64) | 2（2.22） |  |
| **AST/ALT, median** | 1.48 | 1.5 | 0.555 |
| **Cr/** **CysC*100, Mean ± SD** | 64.13±18.89 | 67.91±17.01 | 0.074 |
